# Application of magnetic resonance imaging-related techniques in the diagnosis of sepsis-associated encephalopathy: present status and prospect

**DOI:** 10.3389/fnins.2023.1152630

**Published:** 2023-05-25

**Authors:** Shuhui Wu, Yuxin Wang, Yaqin Song, Hongjie Hu, Liang Jing, Wei Zhu

**Affiliations:** ^1^Department of Emergency Medicine, Tongji Hospital, Tongji Medical College, Huazhong University of Science and Technology, Wuhan, Hubei, China; ^2^Department of Intensive Care Medicine, Tongji Hospital, Tongji Medical College, Huazhong University of Science and Technology, Wuhan, Hubei, China; ^3^School of Biomedical Engineering, Guangzhou Medical University, Guangzhou, Guangdong, China

**Keywords:** sepsis-associated encephalopathy, magnetic resonance imaging, imaging diagnosis, sepsis, cognitive impairment

## Abstract

Sepsis-associated encephalopathy (SAE) refers to diffuse brain dysfunction secondary to systemic infection without central nervous system infection. The early diagnosis of SAE remains a major clinical problem, and its diagnosis is still exclusionary. Magnetic resonance imaging (MRI) related techniques, such as magnetic resonance spectroscopy (MRS), molecular MRI (mMRI), arterial spin-labeling (ASL), fluid-attenuated inversion recovery (FLAIR), and diffusion-weighted imaging (DWI), currently provide new options for the early identification of SAE. This review collected clinical and basic research and case reports related to SAE and MRI-related techniques in recent years, summarized and analyzed the basic principles and applications of MRI technology in diagnosing SAE, and provided a basis for diagnosing SAE by MRI-related techniques.

## 1. Introduction

Sepsis is one of the major diseases that seriously endanger human health. Of 48.9 million sepsis patients, about 11 million died in 2017 worldwide, accounting for 19.7% of the total global deaths in that year (Schuler, 2018). Therefore, sepsis is the focus of scholars’ attention. The brain is an important target organ of sepsis, and sepsis-associated encephalopathy (SAE) can lead to multiple neurological dysfunctions and increase the mortality rate in septic patients (Schuler, 2018; Mazeraud et al., 2020). An international survey (Prescott and Angus, 2018) shows a 3-fold increase in prevalence of moderate to severe cognitive impairment (from 6.1% before hospitalization to 16.7% after hospitalization). Meanwhile, sepsis survivors have typical psychiatric disorders, manifesting as depression, anxiety, and post-traumatic stress disorder (PTSD), with incidences of 67%, 49%, and 46% within 24 h of intensive care unit (ICU) discharge, respectively (Calsavara et al., 2021). Cognitive function and mental impairment after sepsis seriously affect the health status and quality of life of sepsis survivors and cause great damage to society, families, and individual patients. Therefore, early interventions and identification of SAE can effectively reduce the incidence of sepsis-related neurological dysfunction and improve clinical prognosis.

Currently, the commonly used clinical examination methods, including computed tomography (CT), electroencephalography (EEG), and biomarkers, play a role in diagnosing SAE, but there are also some limitations. For example, most current neuroimaging studies involving SAE have shortcomings such as small sample size, poor research design, improper measurement methods, or failure to consider confounding variables, which need further investigation (Atterton et al., 2020). Global white matter changes on CT are detected in adult SAE, but CT cannot detect early microscopic lesions in the brain of SAE patients (Stubbs et al., 2013). EEG has been used alone to evaluate the EEG activity in septic patients (Pantzaris et al., 2021), but it is not suitable for screening because of the expertise required for its interpretation. Also, EEG cannot reliably assess fluctuations in the SAE course (Atterton et al., 2020). Studies have shown that biomarkers are of limited value in SAE because the development of SAE is not preceded by biomarker changes and cannot be used to predict the risk of SAE (Simons et al., 2018). Thus far, reliable and easily identifiable biomarkers that are also cost-effective have not been identified (Toft et al., 2019). A growing number of studies have found that magnetic resonance imaging (MRI) related techniques play a major role in diagnosing SAE. Studies have found that up to 70% of patients with clinically confirmed SAE may have abnormal MRI manifestations, including ischemia, leukoencephalopathy, angioedema, and cerebral atrophy. However, there is still a lack of systematic reports on the application and value of MRI-related techniques in diagnosing SAE.

Therefore, this review collected the clinical and basic research and case reports related to SAE and MRI-related techniques in recent years and summarized the current status and prospect of MRI-related techniques application in SAE diagnosis to provide more research basis for diagnosing SAE by MRI-related techniques.

## 2. Sepsis-associated encephalopathy

Sepsis-associated encephalopathy is one of the important encephalopathies demanding greater attention in the ICU. The incidence of SAE in septic patients is about 70%, and the related mortality rate is 56.1% compared with 35.1% mortality in septic patients without SAE (Zhao et al., 2021). SAE survivors exhibit long-term or permanent neurological dysfunction after discharge, including abnormal emotional behavior and cognitive dysfunction, significantly reducing their quality of life, affecting their recovery, and even causing premature death (Feng et al., 2019).

Sepsis-associated encephalopathy pathogenesis is complex. It is currently believed that various pathophysiological mechanisms, such as blood–brain barrier (BBB) dysfunction, oxidative stress, mitochondrial dysfunction, neuroinflammation, and apoptosis, contribute to SAE development, which severely affects the survival and long-term prognosis of patients with sepsis (Zhao et al., 2021).

In sepsis, cerebrovascular endothelial cells, activated by endotoxins and cytokines, synthesize and release reactive oxygen species (ROS), nitric oxide (NO), and pro-inflammatory factors, resulting in increased BBB permeability and vasogenic brain edema. Inflammatory mediators in the peripheral blood enter the brain and promote neuronal injury and brain edema (Yang et al., 2022). Neuro-oxidative stress plays an important role in sepsis. The imbalance between ROS and antioxidant enzymes in sepsis stimulates a series of lipid peroxidation reactions that inhibit the antioxidant cycle in cells. Oxidative stress leads to mitochondrial dysfunction by altering mitochondrial potentials or electron transport chain activity, causing neuronal apoptosis and organ failure (Gu et al., 2021). Whether it involves BBB destruction or oxidative stress, it ultimately leads to a neuroinflammatory response. The neuroinflammatory response is also one of the pathophysiological mechanisms of SAE that plays a key role in neuronal apoptosis and cognitive impairment. Systemic inflammation produces pro-inflammatory cytokines in the brain and promotes microglia activation, leading to neuroinflammation and, ultimately, neuronal apoptosis in vulnerable brain regions (Catarina et al., 2021).

Although scholars have conducted a series of studies on SAE pathogenesis and acquired some findings, the early diagnosis of SAE is still a major clinical problem, which remains an exclusionary diagnosis (Dumbuya et al., 2023). The clinical manifestations of SAE range from mild consciousness disorders to delirium, deep coma, or seizures and cognitive and emotional dysfunction (Stubbs et al., 2013; Gao and Hernandes, 2021). However, the above-mentioned clinical features are not specific. Commonly used diagnostic techniques include EEG, biomarkers, and cranial CT, which have a limited role in SAE patients with certain presentations. Therefore, it is often necessary to comprehensively analyze clinical symptoms, abnormal manifestations of neuroimaging, and biomarkers. Then, excluding other neurological diseases before diagnosing SAE is required. Currently, there is no specific treatment for SAE, and the latest Surviving Sepsis Campaign guideline has recommended early source control, administration of appropriate antimicrobial drugs, and maintenance of end-organ perfusion. The recommended treatment for SAE includes strengthening sleep management, reducing sound and light stimulation, early activity and exercise, strengthening communication between patients and families, medical staff, and the outside world, and avoiding using benzodiazepine sedatives (Atterton et al., 2020). Therefore, we urgently need to find more effective ways to diagnose and treat SAE early to solve the current diagnostic dilemma.

## 3. Principles and applications of MRI-related techniques in SAE diagnosis

### 3.1. Magnetic resonance spectroscopy

Magnetic resonance spectroscopy uses MR principles to noninvasively quantify metabolite levels in interested tissues. The signal in MRS is generated by applying a specific resonant radiofrequency to atomic nuclei (e.g., 1H) in a static magnetic field. The unique chemical property and environment result in unique proton resonance frequency and peak shape for each metabolite (Pasanta et al., 2021).

Magnetic resonance spectroscopy can be used to identify changes in more than 20 metabolic compounds in brain injury areas (Bartnik-Olson et al., 2021). The relative levels of N-acetyl aspartate (NAA), creatine (Cr), and choline (CHO) are particularly associated with SAE. NAA is a sign of complete metabolic function in neuronal mitochondria. High NAA concentrations in healthy neurons reflect neuronal density. A decrease in NAA level indicates neuronal loss or dysfunction and predicts the onset of neuronal injury (Bartnik-Olson et al., 2021). Cr is composed of phosphocreatine and its free precursor, which is related to brain energy metabolism (Bartnik-Olson et al., 2021). Due to mitochondrial dysfunction in sepsis patients (Manfredini et al., 2019), abnormal brain energy metabolism can affect Cr levels. Increased CHO is associated with membrane synthesis, increased cell numbers, and repair of neuronal damage (Wen et al., 2017). Conversely, the decrease in CHO levels can indirectly predict cell death. Thus, all three metabolic compounds are markers of neuronal apoptosis, which is an important pathophysiological mechanism leading to SAE. After neuronal apoptosis due to mitochondrial dysfunction, insufficient production of adenosine triphosphate (ATP), impaired brain metabolism, cell membrane cleavage, and other pathological processes, the levels of NAA, Cr, and CHO and their mutual ratio are changed. Apart from that, Myo-inositol (mI) indicated microglial-induced neuroinflammation in the brain (El-Abtah et al., 2022). As mentioned previously, systemic inflammation produces pro-inflammatory cytokines in the brain and promotes microglia activation, leading to neuroinflammation and, ultimately, neuronal apoptosis in vulnerable brain regions (Catarina et al., 2021).

Wen et al. (2017) have found that the NAA/Cr ratio was significantly decreased in the SAE group by MRS, and the NAA/Cr ratio correlated well with the apoptosis rate in brain tissue with a absolute value of r-value as high as 0.925. Bozza et al. (2010) have reported an approximately 40% reduction in the NAA/CHO ratio in SAE animals 6 h after cecum ligation and puncture (CLP) compared with the control group. Towner et al. (2018) have found that the ratio of NAA/CHO, and Cr/CHO in the brain of SAE rats significantly decreased at different periods. However, in SAE animal models, the metabolite ratio did not significantly decrease at 24 h after lipopolysaccharide (LPS) injection. This apparent difference might be the result of different experimental models. Li et al. (2022) have found that NAA/Cho ratio in SAE animals decreased at both 7 and 14 days after CLP surgery, which is consistent with previous findings. In addition to this, mI/Cr and Glx/Crb ratios were significantly increased on days 7 and 14 after CLP. It has been shown that mI can cause neuroglial cell differentiation and the elevation of mI/Cr ratio is often interpreted as a reflection of neuroglial cell activation (Schnider et al., 2020); the elevation of Glx/Crb can be interpreted as hippocampal osmotic dysregulation and astrocyte swelling (Karczewska-Kupczewska et al., 2018). This suggests that mI/Cr and Glx/Cr ratios are important biomarkers in the CLP-induced SAE rat model (Li et al., 2022) and that MRS can be used for early diagnosis of SAE.

Therefore, MRS can timely respond to the status of neuronal damage by monitoring level changes of metabolites in the brain tissues of sepsis patients. Thus, we can diagnose SAE at an early stage. At present, MRS is more frequently used in basic research but has not been applied in clinical diagnosis on a large scale. However, in terms of the high correlation between the NAA/Cr ratio and neuronal apoptosis obtained from animal studies, MRS is a powerful tool for future diagnosis of SAE.

### 3.2. Molecular magnetic resonance imaging

Free radicals in SAE patients may be detected by combining the immuno-spin trapping (IST) technique with mMRI (Towner et al., 2018). A spin-trapping compound, 5,5-dimethyl-pyrroline-N-oxide (DMPO), is used to trap and stabilize free radicals generated during oxidative stress. An anti-DMPO probe, is also used. The anti-DMPO probe normally does not permeate BBB and cannot be visualized in the brain tissue. In SAE, the intravenously injected anti-DMPO probe can permeate the brain tissue for visualization due to BBB disruption. Anti-DMPO probes can bind antibodies against DMPO radical adducts and MRI contrast agents, and a contrast agent (Gd-DTPA), as a component of MRI signal transduction, can increase the signal intensity of MRI (Mason, 2016; Towner et al., 2018), which can ultimately target free radicals in brain tissue (Towner and Smith, 2018).

Oxidative stress is one of the important pathophysiological mechanisms of SAE, and free radicals are important products of the oxidative stress response in sepsis. In animal models of SAE, oxidative stress response exists in different brain regions, and excessive ROS are detected in different brain tissue, inducing ultrastructural damage to the mitochondria, mitochondrial dysfunction and brain injury (Catalao et al., 2017; Wu et al., 2019). Therefore, the detection of free radical concentration in the brain using free radical-targeted mMRI has contributed to the early inference of oxidative stress response and mitochondrial dysfunction. Meanwhile, the diffusion and distribution of free radicals in the brain can be clearly and intuitively observed by detecting the anti-DMPO probe, which helps clarify the disturbance of BBB in the brain. In summary, mMRI is an important technique for the early diagnosis of SAE.

Towner et al. (2013) have shown that the levels of free radicals were increased in the brain, liver, and lungs of sepsis rats by mMRI. A follow-up study has demonstrated that mMRI revealed a significant increase in the level of free radicals trapped in SAE rats’ brains at 24 h and 1 week after LPS injection compared to the control group (Towner et al., 2018). All these results emphasize that the levels of free radicals and ROS are altered in SAE patients. On the other hand, Towner has found that in the control group, intact BBB in normal mice did not allow probes to enter brain tissue, but in the brain of SAE mice, the anti-DMPO probes were distributed diffusely. It is inferred that the areas of increased mMRI signal can localize the site of brain injury and determine its severity.

To sum up, free radical-targeted mMRI is a non-invasive way to detect free radical levels in the patient’s brain through MRI signal intensity percentage (SI%) and T1 change percentage (%T1). It can determine impaired mitochondrial function, as well as assist in judging increased BBB permeability and localizing areas of brain damage, as a way to diagnose the onset of SAE. Although the above-mentioned studies only involved animal experiments, with the gradual maturity of mMRI technology, clinical applications are also in further development. Besides, mMRI can also play a significant role in drug and post-intervention curative effect research, providing a breakthrough to understanding SAE pathogenesis, early diagnosis of SAE, and post-intervention efficacy assessment through imaging technology.

### 3.3. Arterial spin-labeling

Arterial spin-labeling is an MRI technique that uses arterial blood as an endogenous tracer to noninvasively visualize blood flow (Muir, 2018). The most common use of ASL is brain perfusion imaging. The blood is labeled before entering the brain through the carotid and vertebral arteries, reducing blood magnetization intensity in the brain. As a result, the images collected downstream of the marker positions appear slightly darker. The difference obtained by subtracting the labeled image from the control is approximately proportional to the perfusion rate and volume of *blood* entering the brain since the marker can be calculated (Hernandez-Garcia et al., 2019). Since the signal of magnetically labeled blood decays relatively quickly, physicians can perform multiple blood flow measurements using ASL, allowing dynamic measurements or averaging *multiple results* to improve the signal-to-noise ratio (Muir, 2018).

Arterial spin-labeling can be used to monitor changes in cerebral blood perfusion in real time, which is a key mechanism in SAE development. In sepsis, the decrease in cerebral perfusion may be induced by neuroinflammatory responses, hypocapnia, and endothelial dysfunction. Abnormal cerebrovascular self-regulation mechanism leads to secondary ischemia and hypoxia injury, subsequently causing brain cell dysfunction and poor neural recovery. Additionally, sedatives can also alter cerebrovascular reactivity and significantly reduce cerebral blood flow (CBF; Fabio Silvio Taccone SSFF, 2013). Therefore, ASL helps physicians monitor the CBF of septic patients in real time, detect the decrease in perfusion, cerebral ischemia, and hypoxia injury in the early stage, and judge the integrity of cerebrovascular self-regulation mechanisms for early diagnosis of SAE.

A study has found that the ASL results showed that the CBF in the cerebral cortex of rats in the SAE group increased and then significantly decreased over time compared to the control group, but the CBF in the thalamus significantly increased, while the CBF in the hippocampus significantly decreased 6 weeks after LPS exposure (Towner et al., 2018). The ASL results of another study have shown that the blood flow distribution in the cerebral cortex was significantly reduced in the SAE group compared with the sham surgery group, but there was no change in other regions (Griton et al., 2020). Another study has confirmed that ASL results showed that the CBF of vasopressor-dependent patients with sepsis was 62% higher than that of the control group when mean arterial pressure (MAP) reached the target of 65 mm Hg, and the results were consistent across all interested regions (Masse et al., 2018). The above-mentioned studies had opposite conclusions, which can be attributed to the following points. First, the onset time and severity of hypotension and image acquisition time were different. Septic shock initially reduces CBF, followed by reactive hyperperfusion after resuscitation and stabilization. Second, these controversial conclusions may be related to different models and research species. Finally, the different choices of vasopressors and sedation may contribute to the differentials. On the other hand, the above-presented studies have shown that most SAE patients would have significant changes in CBF. Therefore, ASL can monitor the changes in CBF in septic patients in real-time, capturing the changes in CBF and making it possible for early diagnosis of SAE. However, there is no unified conclusion on the early changes of CBF in SAE patients. Moreover, more systematic experiments are needed.

Cerebral blood flow impact factors are MAP, intracranial pressure, and autonomic regulation of cerebral vessels. Therefore, monitoring CBF by ASL during sepsis can timely grasp information on the level of CBF in septic patients, dynamically evaluate whether CBF can adapt to the current pathophysiological state, and speculate the severity of illness and brain function. Besides, medical staff can speculate the integrity of cerebrovascular self-regulation mechanism in combination with their MAP and intracranial pressure and adjust the dose and timing of sedatives and vasopressin. It is vital to avoid SAE and secondary cerebral ischemic events in patients.

### 3.4. Fluid attenuated inversion recovery

**Figure 1:**
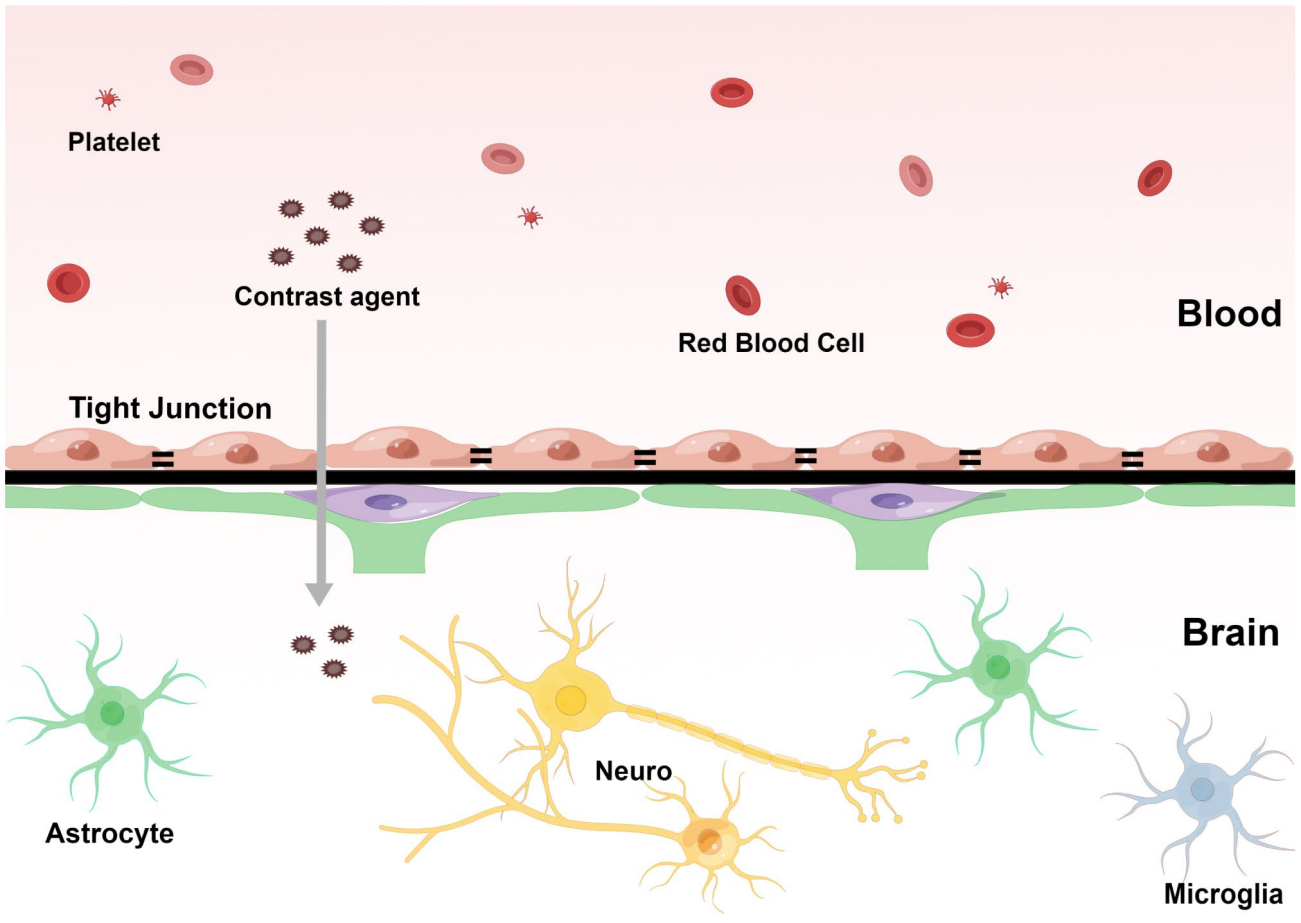
The contrast agents pass through the blood–brain barrier and penetrate the capillaries into the extravascular cellular space. The direction of infiltration is shown by the arrow. (By Figdraw, http://www.figdraw.com).

Fluid attenuated inversion recovery is a special inversion recovery pulse sequence that can effectively display the area of T2 extension and suppress the cerebral spinal fluid (CSF) signal (Ahn et al., 2022). Additionally, contrast-enhanced FLAIR may provide information that is not available in T1-weighted contrast-enhanced MRI for detecting brain diseases, and its imaging principle is closely related to the disruption of central nervous system barrier structures (Ahn et al., 2022). In SAE, disrupted BBB may cause contrast agents to leak from the blood and be diluted by the surrounding liquid (Figure 1). Due to the local shortening of the T1 relaxation time caused by the leakage of the contrast agent into the CSF, the CSF is no longer fully suppressed by the inversion pulse and presents as a high signal (Freeze et al., 2020). The enhanced CSF signal on contrast-enhanced FLAIR can be used to determine that the extravasation of the contrast medium is caused by BBB disruption. Therefore, white matter hyperintensity (WMH) in the contrast-enhanced FLAIR imaging may suggest BBB disruption.

Blood–brain barrier destruction plays an important role in SAE development, leading to neural cell damage, brain edema, and accumulation of blood-derived inflammatory mediators in the brain, resulting in brain dysfunction in patients with sepsis (Zhao et al., 2021). Therefore, FLAIR imaging can provide a new perspective on detecting BBB destruction in SAE patients. It can determine the integrity of BBB by FLAIR and contrast-enhanced FLAIR visualization of brain tissue and the area and extent of contrast agent leakage by a high signal of brain parenchyma to improve the early diagnosis rate of SAE.

**Figure 2:**
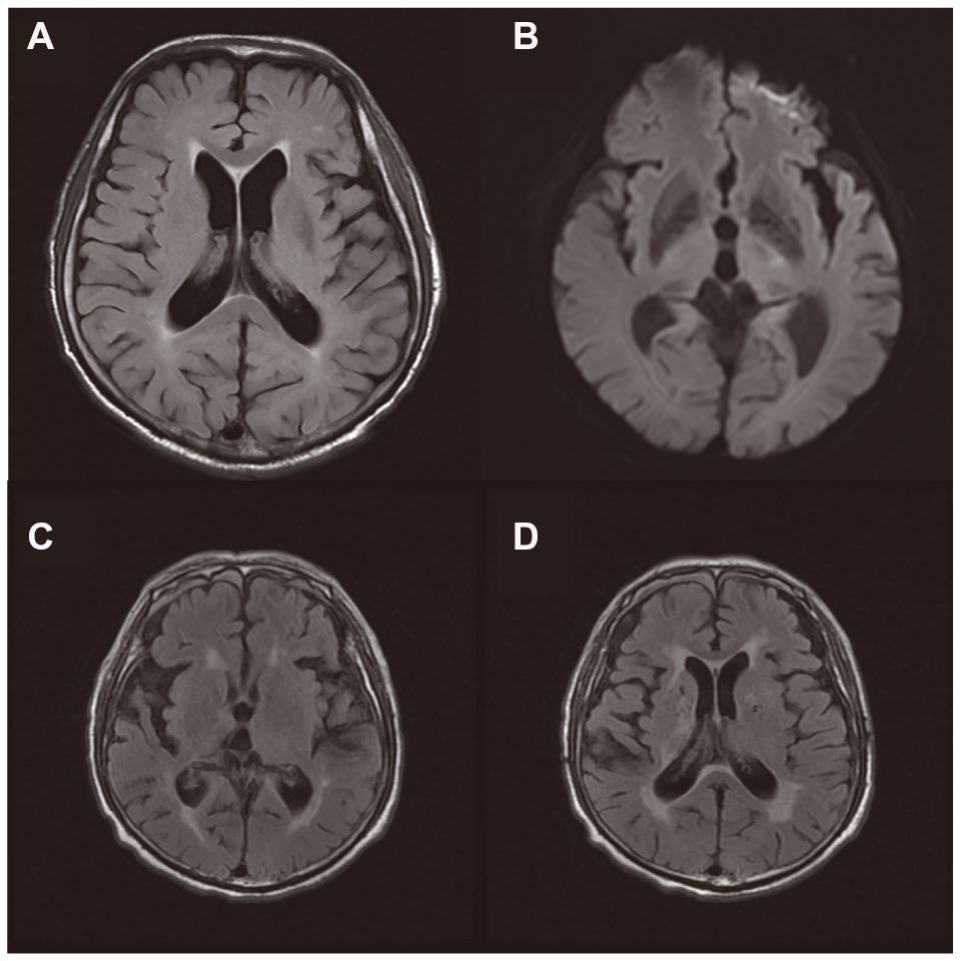
Brain magnetic resonance imaging of two patients during Sepsis-associated encephalopathy (SAE). **(A,B)**: An 75-year-old male patient with serious pneumonia was diagnosed with SAE because of respiratory infection. MRI on day 9 showed high signal on T2-FLAIR sequence, with speckled and patchy WMH in bilateral periventricular cerebral white matter (grade 2 leukoencephalopathy). DWI showed small patchy slightly high signal in the left thalamus region. **(C,D)**: An 79-year-old male patient with urosepsis and cystostomy was diagnosed with SAE. T2-FLAIR sequence showed increased signals in the pontocerebrum, bilateral basal ganglia, radial crown, centrum semiovale, and bilateral frontotemporal parietal lobes.

Currently, there are few systematic studies of SAE using FLAIR. We summarized 19 cases of SAE diagnosed by FLAIR (Finelli, 2004; Kondo et al., 2009; Piazza et al., 2009; Ehler et al., 2017; Shindo et al., 2018). Among them, the positive rate of FALIR examination in SAE patients was up to 78.9%, and most of them could identify WMH manifestations, in which the signal distribution could be punctate, patchy, fused, and diffuse (Table 1). In an MRI study of infectious shock, a high percentage of patients with infectious shock had early brain imaging abnormalities, accounting for about 75%. Approximately 56% of patients had lesions characterized by high intensity on FLAIR, showing varying degrees of white matter lesions, mainly distributed within the Virchow-Robin space, ranging from multiple small areas to diffuse lesions, and the white matter lesions worsened with increasing shock duration (Figure 2). FLAIR findings were also associated with patient prognosis. Patients with normal manifestations survived without neurological sequelae, while non-survivors had severe brain lesions on MRI (Sharshar et al., 2007).

**Table 1:** Nineteen cases of sepsis-associated encephalopathy (SAE) diagnosed by fluid-attenuated inversion recovery (FLAIR).

Author (Year)	Research summary	Age/sex	MRI-FLAIR lesion	Outcome
Kondo et al. (2009)	Case report: two children with SAE, seizures and prolonged coma serial neuroimaging performed	2y9m/F	Blurred corticomedullary junction and narrowed cortical sulci	Brain death on day 73
		17 m/M	Extended WMH	Severe disability after 1.5 years
Shindo et al. (2018)	Case report: A 66-year-old man with SAE characterized by prolonged fever and diarrhea.	66y/M	WMH	Severe Disability on Day 60
Piazza et al. (2009)	Prospective study of 4 ICU patients with sepsis Outcome measures: MRI and S100B levels	51y/M	FLAIR and T2-hyperintense foci in the frontal regions	Recovery on day 90
		61y/M	FLAIR and T2-hyperintense foci in the periventricular regions	Died 4 days later
Finelli (2004)	Case report: SAE post renal transplantation MRI imaging.	48y/F	FLAIR signals increased in the bilateral basal ganglia, cerebellum, brainstem, and temporal lobe	Death on Day 13
Ehler et al. (2017)	Prospective study of 13 patients with sepsis who had clinical features of SAE for two distinct patterns of neuroaxonal injury in sepsis	63y/F	Diffuse WMH	Dead
		82y/F	Diffuse WMH	Survival on day 100
		73y/M	None	Survival on day 100
		57y/M	None	Survival on day 100
		55y/M	Patchy/confluent WMH	Survival on day 100
		80y/F	Punctiform WMH	Dead
		44y/M	None	Survival on day 100
		76y/F	Punctiform WMH	Dead
		74y/F	Patchy/confluent WMH	Survival on day 100
		75y/M	Diffuse WMH	Survival on day 100
		54y/F	None	Survival on day 100
		60y/F	Punctiform WMH	Survival on day 100
		81y/M	Patchy/confluent WMH	Survival on day 100

The high positive rate of FLAIR indicates that FLAIR is an effective tool to improve the early diagnosis of SAE and is closely related to prognosis, which can be used to assess the effect of interventions and infer the long-term prognosis. When FLAIR shows typical abnormalities, such as WMH, it can be presumed that patients with sepsis have an accumulation of inflammatory mediators in the brain. Thus, we can consider the possibility of developing brain dysfunction. However, WMH is also a typical manifestation of aging and cerebrovascular diseases (Dounavi et al., 2022), which may be caused by many different pathologies, and is not specific enough to be used alone to diagnose SAE. When diagnosing SAE, combining it with other imaging diagnostic techniques is necessary.

### 3.5. Diffusion-weighted imaging

Diffusion-weighted imaging is a non-invasive MR technique for measuring the dispersion of water in the brain (Messina et al., 2020). The Apparent Diffusion Coefficient (ADC) is a quantitative index calculated from DWI to quantify the diffusing capacity of water in tissues (Beig Zali et al., 2021). DWI reflects whether the activity of water is restricted, showing a high or low signal if water activity is restricted or not, respectively. ADC reacts to the degree of water dispersion, and the larger the ADC value, the greater the degree of dispersion.

When angiogenic edema occurs, plasma leaks into the extracellular space. The diffusion movement of extracellular water is relatively free, the DWI shows low signal intensity, and the ADC value is higher compared to normal brain tissue. When *cytotoxic* edema occurs, extracellular water enters the cell, and intracellular water is restricted by the membrane structure, with their diffusion movement becoming limited. Meanwhile, the extracellular gap is narrowed due to cell swelling, water diffusion is bounded, and DWI shows a high signal while the ADC value decreases. Vasogenic edema and cytotoxic edema are important processes in SAE development, which can reflect the BBB disruption and cell dysfunction in SAE. Therefore, according to the signal level of DWI and ADC, we can detect water movement and diffusion, indirectly identify vasogenic edema and cytotoxic edema in brain tissue, and further infer the destruction of the BBB; hence, SAE can be diagnosed at an early stage.

A study has shown that brain tissue ADC was diffusely decreased in SAE animals compared to the control group. Furthermore, the decline in ADC was more pronounced in the animals who died after CLP. At the same time, ADC diffusion is enhanced in areas of high T2-weighted intensity at the base of the brain (Bozza et al., 2010). This suggests that there was not only neuronal edema in SAE animals’ brains but also intense fluid accumulation in the area around the circle of Willis. Moreover, intracerebral fluid accumulation was closely related to the survival of mice, which is a reliable indicator of SAE severity. Another study has also confirmed that axial water dispersion was increased in the corpus callosum, while ADC in the cortex and striatum did not change in mice after CLP compared to controls (Griton et al., 2020). T2 hyperintensities in the cortex, striatum, and brain base are considered to be vascular-derived edema rather than cellular edema (AVJB, 1997). This implies that SAE is associated with fluid accumulation in the base of the brain, as indicated by T2-weighted signal enhancement (Griton et al., 2020). Another study has demonstrated that in the early stage (3.5 h) of LPS-induced SAE, there was little change in ADC (Rosengarten et al., 2008). The lack of white matter change could be attributed to short stimulation time or lack of polymicrobial stimulation.

Diffusion-weighted imaging provides subjective description through images, and ADC value is an objective quantitative index to reflect water dispersion. The combination of DWI image and ADC value can infer the existence of brain injury early by showing brain cell edema and intracranial fluid accumulation and further provide information for the diagnosis of SAE. According to the difference in the magnitude of ADC changes, we can determine the long-term prognosis of SAE patients and ultimately improve the early diagnosis rate of SAE and the accuracy of the severity assessment.

### 3.6. Functional MRI

Functional MRI (fMRI) is a neuroimaging tool utilizing MRI to image dynamic changes in brain tissue caused by neurometabolic changes. Resting-state fMRI (rs-fMRI) detects blood oxygenation level-dependent (BOLD) signals and provides information on regional spontaneous neuronal activity in terms of amplitude of low frequency fluctuations (ALFF), which is the most commonly used analysis method for fMRI (Zou et al., 2008; Huang et al., 2021). Changes in neural activities may appear when subjects perform specific cognitive tasks or spontaneously when subjects are unconscious (“resting state”; Chen and Glover, 2015). Currently, fMRI has become a powerful tool for the non-invasive assessment of human brain activity and function, allowing accurate localization of activated functional brain areas. fMRI plays an important role in the diagnosis of encephalopathies, and the rs-fMRI can also evaluate the functional connectivity in different brain regions. A study on the changes in hippocampal functional connectivity (FC) in rats with SAE by rs-fMRI has shown that FC in the hippocampus of SAE rats was enhanced and positively correlated with affective impairment. The FC between the hippocampus and other brain regions might be a potential neuroimaging marker for cognitive or psychiatric impairment triggered by SAE (Yao et al., 2022). Another study of the default-mode network (DMN) about different cognitive and affective disorders has found that SAE rats’ functional connectivity between the retrosplenial cortex and medial prefrontal cortex is increased. The conclusion is consistent with the above-mentioned experiment. Moreover, FC within the DMN in rats with other psychiatric disorders, for example, PTSD, was significantly different from the SAE rats (Ji et al., 2018). This suggests that the changes in FC revealed by rs-fMRI might be helpful in the early diagnosis of SAE.

Li et al. (2022) measured the ALFF value changes in the rrats’ hippocampus at 7 and 14 days post CLP surgery. They found that the ALFF values in the right CA-1 area of the hippocampus was higher at day 7 post CLP surgery than controls, and lower at 14 days post CLP surgery. Furthermore, ALFF value of right CA-1, was negatively related to cognitive function, suggesting brain ALFF values and metabolite alterations can be used to evaluate cognitive deficits in SAE rats. The ALFF values of the right CA-1 area are an excellent biomarker to evaluate the cognitive function in SAE rats.

Therefore, fMRI might be an important tool for the early identification and prediction of encephalopathy, as well as neuronal functionality changes induced by drugs and rehabilitation strategies.

### 3.7. T1 weighted image, diffusion-tensor imaging

The T1-weighted phase and the T2-weighted phase are the most frequently used sequences in MRI. Usually, they can detect many lesions in brain, showing different gray levels in the image, but it is indeed difficult to distinguish them from tumors, small-vessel ischemia, microhemorrhages, epilepsy lesions, cerebral infarction, white matter injury and other lesions. The normal structural MRI technique cannot diagnose SAE independently.

Diffusion-tensor imaging (DTI) is a noninvasive MRI technique that has mostly been used to evaluate microstructural changes in the brain by measuring the motility of water molecules in tissue (Tae et al., 2018) and use the characteristics of anisotropy to track the white matter fiber tracts. Currently, DTI has been used in the resection of peripheral nerve tumors, Parkinson’s disease, Alzheimer’s dementia, epilepsy, multiple sclerosis, amyotrophic lateral sclerosis and ischemic stroke. Studies on its use in SAE patients are still limited. The relationship between Neuroinflammation and DTI imaging of the brain in SAE patients remains to be further elucidated.

In conclusion, although MRI-related techniques have a long history and are gaining growing popularity in the clinical evaluation of cognitive impairment such as Alzheimer’s disease, post-traumatic stress disorder, and post-operative cognitive dysfunction (Herringa, 2017; Zhang et al., 2019), only a few studies have applied these techniques for early diagnosis of SAE.

In the long term, with the continuous development of fMRI technology, all these methods may play a significant role in the early diagnosis of SAE, and further research is needed to promote it.

## 4. Conclusion

**Figure 3:**
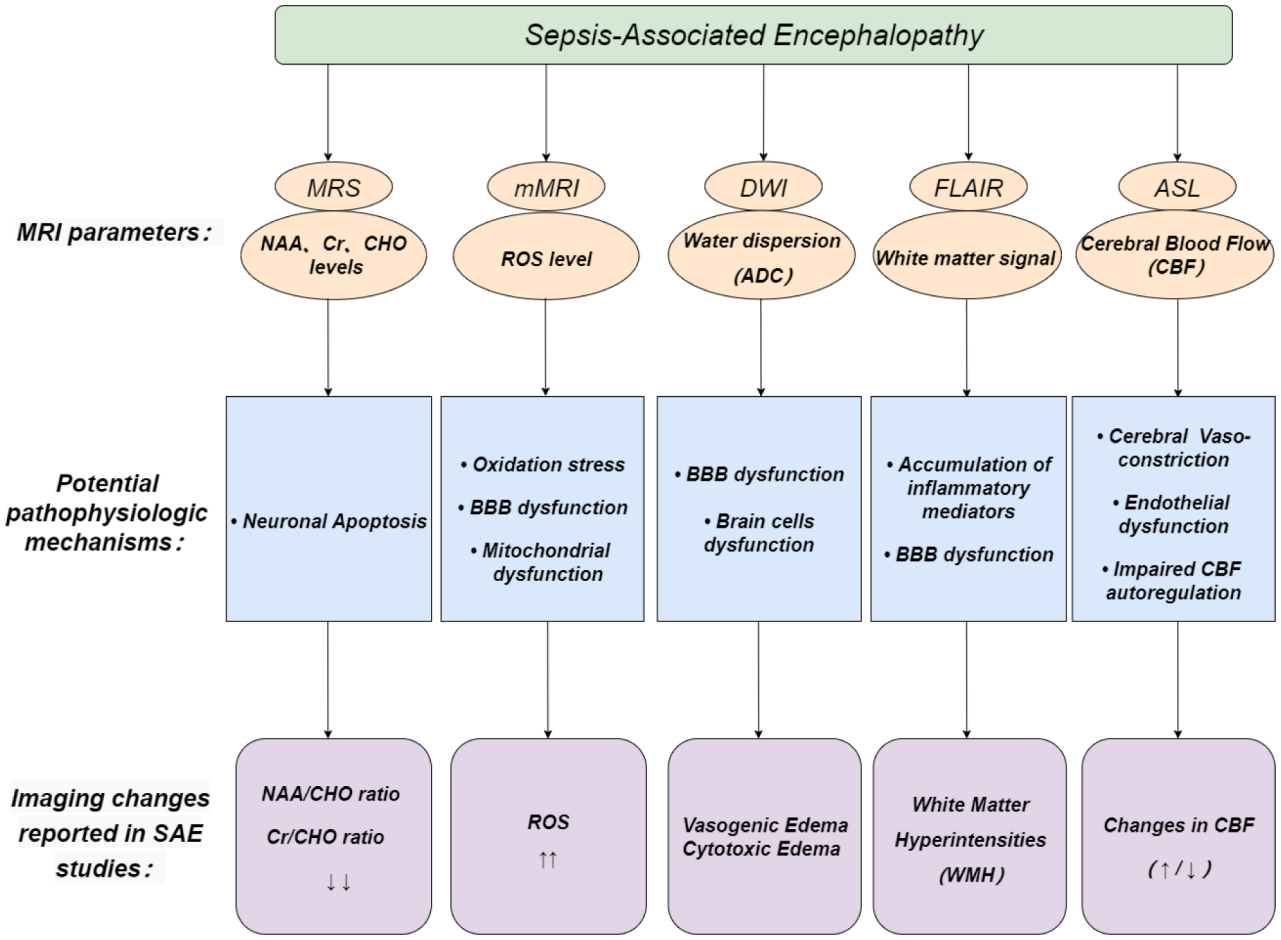
Possible role of MRI-related techniques in the diagnosis of SAE. Pink circles depict MRI parameters, blue boxes depict potential pathophysiologic mechanisms of SAE, and purple boxes depict imaging changes reported in SAE studies.

In this study, we summarized the main principles of multiple MRI related techniques and the experimental and clinical results regarding the MRI-related techniques performance of patients with SAE (Figure 3). MRI-related techniques show high potential in diagnosing SAE. However, the pathogenesis of SAE is multifactorial. Imaging changes mentioned above can also be seen in other encephalopathies, and no single diagnostic technique can cover every aspect of the disease. Therefore, other supplementary imaging methods, such as EEG and head CT, must be used to diagnose SAE accurately. Additionally, we need to keep track of innovative new technologies. The new generation of fMRI technology is still evolving continuously, and its accuracy has greatly improved. These new technologies may play an important role and be applied to the early diagnosis of SAE.

## Author contributions

WZ contributed to the conception and critically revised the manuscript for important intellectual content. SW was involved in drafting and revising the manuscript. YS, HH, YW, and LJ contributed to the data acquisition and classification. SW, YW, YS, HH, LJ, and WZ met authorship criteria. All authors contributed to the article and approved the submitted version.

## Funding

This Project was supported by the Key R&D Program of Hubei Province (grant no. 2022BCA038).

## Conflict of interest

The authors declare that the research was conducted in the absence of any commercial or financial relationships that could be construed as a potential conflict of interest.

## Publisher’s note

All claims expressed in this article are solely those of the authors and do not necessarily represent those of their affiliated organizations, or those of the publisher, the editors and the reviewers. Any product that may be evaluated in this article, or claim that may be made by its manufacturer, is not guaranteed or endorsed by the publisher.

## Abbreviations

SAE: sepsis-associated encephalopathy
MRI: magnetic resonance imaging
MRS: magnetic resonance spectroscopy
mMRI: molecular magnetic resonance imaging
ASL: arterial spin-labeling
FLAIR: fluid-attenuated inversion recovery
DWI: diffusion-weighted imaging
PTSD: post-traumatic stress disorder
CT: computed tomography
EEG: electroencephalography
ICU: intensive care unit
BBB: blood–brain barrier
ROS: reactive oxygen species
NO: nitric oxide
NAA: N-acetyl aspartate
Cr: creatine
CHO: choline
ATP: adenosine triphosphate
mI: myo-inositol
CLP: cecum ligation and puncture
IST: immuno-spin trapping
DMPO: 5,5-dimethyl-pyrroline-N-oxide
SI%: signal intensity percentage
%T1: T1 change percentage
CBF: cerebral blood flow
CSF: cerebral spinal fluid
WMH: white matter hyperintensity
ADC: apparent diffusion coefficient
fMRI: functional MRI
rs-fMRI: resting-state fMRI
DTI: diffusion tensor imaging
FC: functional connectivity
DMN: default-mode network
BOLD: blood oxygenation level-dependent
T1WI: T1 weighted image
ALFF: amplitude of low frequency fluctuations
